# Electronic tissue compensation achieved with both dynamic and static multileaf collimator in eclipse treatment planning system for Clinac 6 EX and 2100 CD Varian linear accelerators: Feasibility and dosimetric study

**DOI:** 10.4103/0971-6203.33241

**Published:** 2007

**Authors:** Rajesh A. Kinhikar, Pramod K. Sharma, Sachin Patkar, Chandrashekhar M. Tambe, Deepak D. Deshpande

**Affiliations:** Department of Medical Physics, Tata Memorial Hospital, Parel, Mumbai, India

**Keywords:** Dynamic multileaf collimator, electronic tissue compensator, quality assurance, static multileaf collimator

## Abstract

Dynamic multileaf collimator (DMLC) and static multileaf collimator (SMLC), along with three-dimensional treatment planning system (3-D TPS), open the possibility of tissue compensation. A method using electronic tissue compensator (ETC) has been implemented in Eclipse 3-D TPS (V 7.3, Varian Medical Systems, Palo Alto, USA) at our center. The ETC was tested for head and neck conformal radiotherapy planning. The purpose of this study was to verify the feasibility of DMLC and SMLC in head and neck field irradiation for delivering homogeneous dose in the midplane at a pre-defined depth. In addition, emphasis was given to the dosimetric aspects in commissioning ETC in Eclipse. A Head and Neck Phantom (The Phantom Laboratory, USA) was used for the dosimetric verification. Planning was carried out for both DMLC and SMLC ETC plans. The dose calculated at central axis by eclipse with DMLC and SMLC was noted. This was compared with the doses measured on machine with ion chamber and thermoluminescence dosimetry (TLD). The calculated isodose curves and profiles were compared with the measured ones. The dose profiles along the two major axes from Eclipse were also compared with the profiles obtained from Amorphous Silicon (AS500) Electronic portal imaging device (EPID) on Clinac 6 EX machine. In uniform dose regions, measured dose values agreed with the calculated doses within 3%. Agreement between calculated and measured isodoses in the dose gradient zone was within 3 mm. The isodose curves and the profiles were found to be in good agreement with the measured curves and profiles. The measured and the calculated dose profiles along the two major axes were flat for both DMLC and SMLC. The dosimetric verification of ETC for both the linacs demonstrated the feasibility and the accuracy of the ETC treatment modality for achieving uniform dose distributions. Therefore, ETC can be used as a tool in head and neck treatment planning optimization for improved dose uniformity.

Missing tissue compensation is of great importance to achieve dose homogeneity in head and neck radiotherapy (RT). Different strategies of tissue compensation have been reported.[1] The multileaf collimator (MLC) has played a vital role in this aspect.[2–6] The features of dynamic multileaf collimator (DMLC) and static multileaf collimator (SMLC), along with three-dimensional treatment planning system (3-D TPS), open up the possibility of modulating photon beam to create any desired fluence distribution within the radiation field. Through suitable intensity modulation (IM) across the treatment field, the 3-D TPS can design a compensated field that will deliver a uniform and homogeneous dose in the midplane. A method using electronic tissue compensator (ETC) to produce IM fields for missing tissue compensation has been implemented in Eclipse 3-D TPS (V 7.3, Varian Medical Systems, Palo Alto, USA) at our center. The ETC was tested for head and neck conformal RT planning.

The purpose of this study was to verify the feasibility of DMLC and SMLC in head and neck field irradiation for delivering homogeneous dose in the midplane at a pre-defined depth. In addition, the emphasis was given to the dosimetric aspects in commissioning ETC in eclipse. Additionally, dosimetric verification and comparison of ETC in phantom on two Varian linear accelerators (Clinac 6 EX and 2100 CD) were performed, and the results are reported.

## Materials and Methods

### Head and neck phantom

A head and neck phantom (The Phantom Laboratory, USA) procured earlier at our center was further modified to accommodate various dosimetric detectors. A special insert for an ion chamber (CC13, Scanditronix Wellhofer, Sweden) was fabricated in the midline of phantom normal to the transverse plane, and the same was used for thermoluminescence dosimetry (TLD) measurements. In addition, a special holder was designed to place the extended dose range (EDR2, Eastman Kodak, Rochester, NY) films in sagittal and coronal planes. The phantom has also the provision to fill water to maintain homogeneous tissue equivalent medium for dosimetry. This phantom was then scanned at 0.2 cm interval in a Somatom CT-simulator (Siemens Oncology, Germany). The scanned images were exported via DICOM to Eclipse.

### Linear accelerators and MLC

We have MLC provision on two linear accelerators. The Clinac 6 EX and 2100 CD linear accelerators have Millennium 120- leaves MLC and 52-leaves MLC respectively. The leaves of these single-focus MLCs have a rounded leaf end. The Millennium MLC includes 60 leaf pairs of which the central 40 leaf pairs cast a shadow of 0.5 cm width at isocenter, and the remaining leaves cast a 1 cm wide shadow. The Clinac 2100 CD has 26 leaf pairs, all projecting a shadow of 1 cm at the isocenter. The MLC transmission specified by the manufacturer was less than 4% for these MLCs. In our setup, the measured interleaf and intraleaf transmission was found to be within 2% and 1.5% respectively for both the linacs. The leaf-positioning accuracy for both the linacs was within 0.1 cm.

### Treatment planning in eclipse

**Figure 1:**
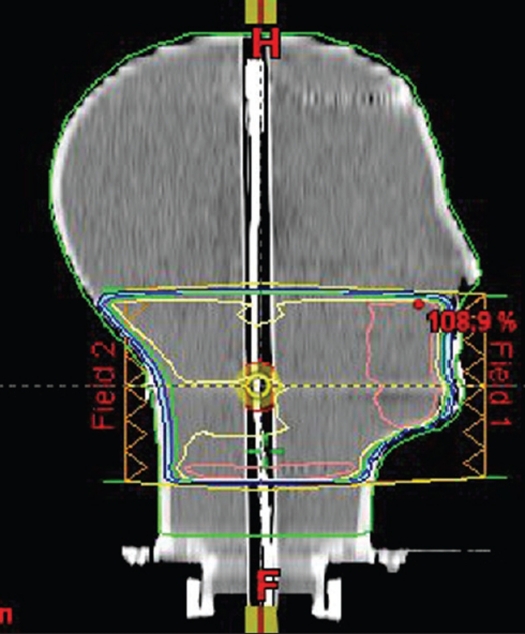
Shows HN phantom with two isocentric, coplanar 6 MV photon beams. The field size was 10 × 10 cm^2^ and the beams were placed anteriorposterior (AP) and posterior-anterior (PA)

Two isocentric, parallel-opposed coplanar 6 MV photon beams with a field size of 10 × 10 cm^2^ were placed anterio-posterior (AP) and posterio-anterior (PA) in HN phantom as shown in Figure 1. The dose (2 Gy) was prescribed at the isocenter (midplane of the phantom). A compensator was attached to each beam so as to achieve a uniform dose of 2 Gy throughout the midplane, and this compensator was then converted to electronic tissue compensator. The electronic compensator is a field modifier implemented by means of the DMLC and SMLC that replaces a mechanical compensator. The Eclipse TPS module produces an optimal modulated field fluence converted into actual fluence by the leaf motion calculator (LMC), which creates a pattern of DMLC and SMLC leaf motion. The compensation method used is planar compensation. The conversion of a standard plane compensator into an electronic compensator creates an optimal fluence, according to calculation parameters. The calculation is done for each field by scanning all points within the field on the selected plane. The compensator calculation begins at the user-defined distance from the edge of the patient tangential border. The optimal fluences are converted into deliverable fluences and into a pattern of DMLC leaf motions by LMC. When the plan and the DMLC motions are completed, the DMLC leaf motion file can be exported to the treatment unit. The uniform dose distribution is computed using the dose back-projection method, in which the optimal fluences are defined. The dose back-projection is done in the diverging field coordinate system.

Electronic compensation is available for open coplanar photon fields. One cannot add an electronic compensator to an electron field, and electronic compensators cannot be used for arc fields or mixed field types (both isocentric and fixed SSD fields). Moreover, one field may not contain both a physical plane compensator and an electronic compensator.

The computational grid size of 2.5 × 2.5 × 2 mm^3^ was used for dose calculation. Isodose distribution was computed using pixel-to-pixel modified Batho inhomogeneity corrections. Dose profiles along the two major axes for central axis were generated with DMLC. Similar procedure was followed to achieve uniform dose distribution in the midplane with SMLC. For SMLC, nine intensity levels were used. Altogether, a total of four plans (two each for Clinac 6 EX and 2100 CD) were generated. The doses calculated at the isocenter by Eclipse with DMLC and SMLC were noted. The calculated doses in the coronal plane of the phantom were then exported to OmniPro™ IMRT film dosimetry system (V 1.4.1 Scanditronix Wellhofer, Germany) for comparison with the actual delivered fluence (measured).

### Dosimetric verification

Absolute central axis dose was measured at the isocenter at the depth of midplane with 0.13 cc thimble ionization chamber and NE electrometer (NE Technology, Essex, UK) in both the linacs under same set-up as in eclipse. The absolute dosimetry with ion chambers was carried out using TRS-398 dosimetry protocol.[7] Verification for doses of intensity-modulated fields was performed to ensure that the doses calculated by the commercial treatment planning system and those administered to the patient were in agreement. Measurements were carried out for both DMLC and SMLC plans.

These measurements were repeated with TLD chips (lithium fluoride doped with magnesium and titanium, LiF:Mg, TI, dimensions 1/8 × 1/8 × 0.035 in.). The TLD chips were irradiated in the midplane of the phantom at isocenter. The TLD measurements were performed on a commercial TLD-reader system (REXON Model UL-320 reader, Ohio, USA) after 24 h after irradiation, and the average of the readings was estimated. For this, we assume our TLD as a benchmark with an estimated accuracy of ± 3%. TLD-100 (quantity 100) chips were annealed carefully at 400°C for an hour and subsequently cooled down to room temperature before using it for clinical purpose. These TLDs were irradiated after 24 h with Co-60 teletherapy machines with a known dose of 2 Gy at 5 cm depth in water-equivalent medium. These TLDs were read carefully using standard method mentioned above. Irrespective of the high variation in the sensitivity of an individual TLD in the entire group of 100, the segregated sensitivity variation in an individual TLD batch (approximately 20-30) was found to be ± 3%. Since the TLDs were read between 24 and 48 h post-irradiation, with proper handling no corrections were required and hence no corrections were applied. Measured doses with the ion chamber and TLD at central axis were then compared with calculated doses from eclipse TPS.

Isodose distributions were also determined by EDR2 radiographic films. The film was kept inside the phantom in the midplane. The measured fluences in the form of isodose curves were then compared with the eclipse-calculated isodose curves. Similarly, the profiles measured on the EDR2 film were compared with those calculated by eclipse. Gamma analysis was performed with 3% and 3 mm as the pass criteria. The gamma histogram was generated and analyzed.

## Results and Discussion

Dose profiles along the two major axes passing through central axis were found to be flat across the treatment field for both DMLC and SMLC plans. These dose profiles were then compared with the profiles from Amorphous Silicon (AS500) electronic portal imaging device (EPID) on the Clinac 6 EX machine.

**Figure 2:**
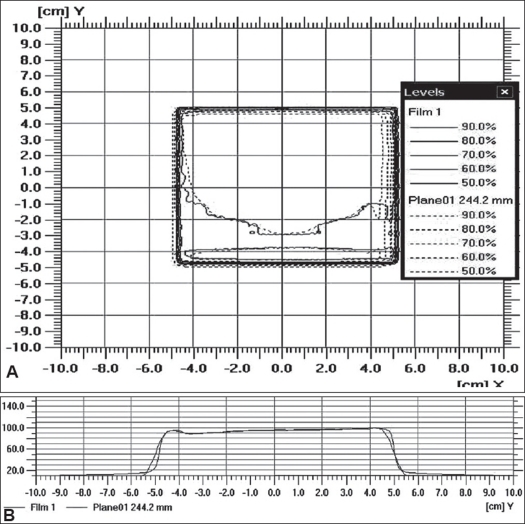
A) Comparison of measured and calculated isodoses. The solid line and the dotted line depict the dose from film and plane (Eclipse) respectively, B) The comparison of measured and calculated profile. The red and green curves show profile from film and plane (Eclipse) respectively

Table 1 shows the dosimetric verification carried out with both the ion chamber and TLD. The results are displayed for ETC delivered with both DMLC and SMLC. In uniform-dose regions, measured dose values (by ion chamber and by TLD) were in close agreement with the calculated doses, viz., within 3%. Agreement between calculated and measured isodoses in the dose gradient zone was within 3 mm. Figure 2A shows the comparison of measured and calculated isodoses. The solid line and the dotted line depict the dose from film and eclipse respectively. The isodose lines of 50, 60, 70, 80 and 90% were compared. Similarly, Figure 2B shows the comparison of measured and calculated profiles in the sagittal plane. The red and the green curves show profiles from film and eclipse respectively.

**Table 1:** Comparison of measured doses at isocenter with ion chamber and thermoluminescence dosimetry against the calculated doses by eclipse. Planned dose was 2 Gy at the isocenter

	*Clinac 6EX*				*Clinac 2100CD*			
		
	*DMLC*		*SMLC*		*DMLC*		*SMLC*	
				
	*IC*	*TLD*	*IC*	*TLD*	*IC*	*TLD*	*IC*	*TLD*
Measured dose (Gy)	1.98	1.97	1.97	2.04	1.98	2.03	2.02	1.97
Calculated dose (Gy)	2	2	2	2	2	2	2	2
% variation	−1	−1.5	−1.5	2	−1	1.5	1	1.5

DMLC: Dynamic multileaf collimator, SMLC: Static multileaf collimator, IC: Ion chamber

The comparison of isodoses and the profiles shows that they are in good agreement and are thus clinically acceptable. Fgiures 3A and 3B show the gamma analysis and the gamma histogram respectively for 3% and 3 mm criteria. From the histogram, it was seen that for more than 97% of the pixels, gamma was found to be within the accepted value of < 1.

**Figure 3:**
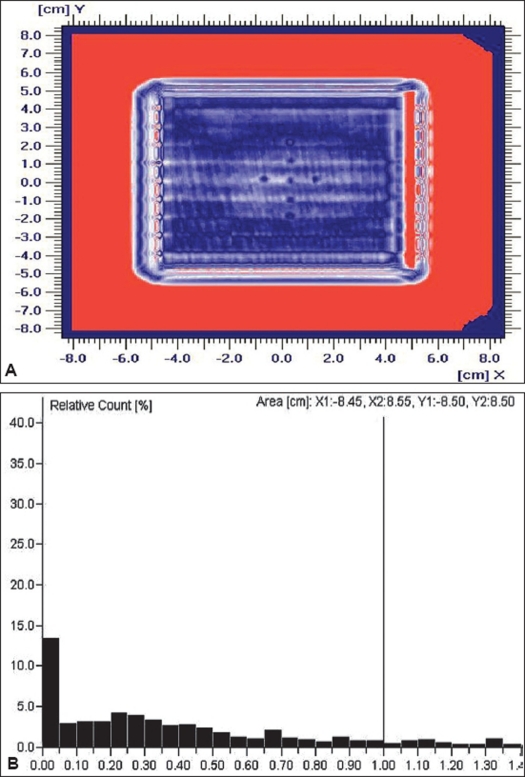
A) Gamma evaluation for 3% and 3 mm criteria, B) Gamma histogram

Usually fixed wedge filters are used for the lateral opposed fields in order to achieve a uniform dose, which could be improved with intensity-modulated fields, taking into account the missing tissue on the entire field area. Moreover, the ETC, compared to physical compensator, can speed up the treatment and also reduce skin dose, since it does not expose the patient to electron contamination. Electronic compensation does not require the high overhead in production that is present in mechanical compensators, and multi-beam treatments are much faster because there is no need to install a different physical compensator before execution of each treatment beam.

For the DMLC-ETC plan, it was noticed that the monitor units (MU) were 1.15 times higher than those of SMLC-ETC plan. This in turn could increase the total treatment time and ultimately the integral dose for the DMLC-ETC plan. But this observation was true only up to the nine intensity levels for SMLC-ETC plan and not for levels higher than that. For more than nine intensity levels, a drastic increase (1.5 times) in MU was observed in SMLC-ETC plan compared to DMLC-ETC plan. However, this was not the scope of our study.

## Conclusion

The dosimetric verification of ETC for both the linacs demonstrated the feasibility and the accuracy of the ETC treatment modality for achieving uniform dose distributions. Flat profiles across the treatment field can be obtained. ETCs provided more flexibility in designing the profiles than the physical compensator method. Further advantages include finer intensity steps, more accurate dose distributions, adjustment during treatments and easy implementation. The use of ETC also eliminates the need for physical trays and mounting slots in the linac treatment head. The time required to design and fabricate the physical compensator can be saved. Therefore, ETC can be used as a tool in head and neck treatment planning optimization for improved dose uniformity. However, since the MLC dynamic mode is involved in the treatment, an extensive quality assurance should be carried out for individual plans.
